# Evaluation of the clinical process in a critical care information system using the Lean method: a case study

**DOI:** 10.1186/1472-6947-12-150

**Published:** 2012-12-21

**Authors:** Maryati Mohd Yusof, Soudabeh Khodambashi, Ariffin Marzuki Mokhtar

**Affiliations:** 1 Center for Technology and Software Management, Faculty of Information Science and Technology, Universiti Kebangsaan Malaysia, Bangi 43600, Malaysia; 2 Anesthesia Department, National Heart Institute, No. 145 Jalan Tun Razak, Kuala Lumpur, 50400, Malaysia

## Abstract

**Background:**

There are numerous applications for Health Information Systems (HIS) that support specific tasks in the clinical workflow. The Lean method has been used increasingly to optimize clinical workflows, by removing waste and shortening the delivery cycle time. There are a limited number of studies on Lean applications related to HIS. Therefore, we applied the Lean method to evaluate the clinical processes related to HIS, in order to evaluate its efficiency in removing waste and optimizing the process flow. This paper presents the evaluation findings of these clinical processes, with regards to a critical care information system (CCIS), known as IntelliVue Clinical Information Portfolio (ICIP), and recommends solutions to the problems that were identified during the study.

**Methods:**

We conducted a case study under actual clinical settings, to investigate how the Lean method can be used to improve the clinical process. We used observations, interviews, and document analysis, to achieve our stated goal. We also applied two tools from the Lean methodology, namely the Value Stream Mapping and the A3 problem-solving tools. We used eVSM software to plot the Value Stream Map and A3 reports.

**Results:**

We identified a number of problems related to inefficiency and waste in the clinical process, and proposed an improved process model.

**Conclusions:**

The case study findings show that the Value Stream Mapping and the A3 reports can be used as tools to identify waste and integrate the process steps more efficiently. We also proposed a standardized and improved clinical process model and suggested an integrated information system that combines database and software applications to reduce waste and data redundancy.

## Background

The application of information technology in healthcare has significant potential and benefits; particularly with regards to innovations in improving both clinical and administrative processes. Health Information Systems (HIS) support specific tasks in the clinical workflow that include order entry, resource planning, accounting, and scheduling [1].

The evaluation of HIS is crucial for ensuring that maximum benefits are gained from the system and for assessing the achievement of its objectives in supporting healthcare delivery services [1]. HIS evaluation analyses a range of factors that may determine its effectiveness, such as change management, user adoption and workflow, and other human and cognitive factors.

Numerous studies on clinical processes have shown that systems often do not fit well with actual clinical practices. Users may refuse to work with a system, if it is not adapted to their routine tasks. Therefore, the implementation of information systems in healthcare is a process of mutual transformation [2]. It has been reported that clinical workflow support is the highest-ranked indicator in the assessment of IT systems in hospitals [3] and has a major determinant effect on HIS adoption [4]. HIS implementation affects the clinical workflow. Therefore, it is essential to evaluate and redesign clinical processes and workflows, to ensure that they fit with the HIS; and thereby achieve a successful implementation [5].

Quality management methods were introduced into healthcare organizations during the 1980s. Over the last decade, these methods have been used to improve the quality of healthcare and its processes. The selection of these techniques depends on multiple factors, such as organizational requirements, objectives and environment, as well as, available resources and knowledge. One of the most popular quality improvement methods, i.e., the Lean method, is designed to improve process efficiency by eliminating non-value-added activities, known as waste [6]. Waste uses resources, but does not add any value to the product or service [7]. The seven most common contextual synonyms of waste are “overproduction, waiting, transport, inappropriate processing, unnecessary inventory, waste of motion, and defects” [8].

A number of studies have reported the application of the Lean method in the analysis of clinical processes. Kuo et al., [9] proposed a new model, known as the Lean Six Sigma System, to improve workflow in a post-anaesthesia care unit. DelliFraine et al., [10] conducted a systematic review to examine the empirical evidence relevant to the Six Sigma and Lean methods, in improving clinical outcomes, care processes, and the financial performance of healthcare institutions. The use of the Lean method, in the management of hip fracture patients, significantly reduced waste and overall mortality and improved patient flow from admission to discharge [11]. In another case, the application of the Lean method in outpatient services increased the capacity for admitting new patients by 27% [12]. The use of the Lean and Six Sigma methods also substantially improved Operating Room (OR) efficiency, in terms of on-time starts and a reduction in the number of cases past 5pm; as well as significant gains in non-operative time, staff overtime, and ORs saved [13].

The Lean method is a good option for optimizing clinical workflow, because it focuses on detailed process components, such as workflow and problems, and then redesigns the processes by removing waste [8]. The Lean method eliminates unnecessary intermediate steps such as time and personnel, and retains only those that add value [14]. Value is created when wasteful activities are removed or reduced. Value can also be increased by adding services and features that are based on customer needs. This aim could also be achieved by shortening delivery cycles or reducing delivery batches without incurring additional costs [8].

Tools and techniques are available that enhance the Lean concept, such as Value Stream Mapping (VSM) and A3 reports [7,8,15-20]. These are clear and objective communication tools that are able to capture workers’ knowledge of work processes in the value stream. When value stream maps are drawn, and A3 documents are written, all specialists and staff can review them. This allows for cross-departmental sharing of process changes and generates even more problem-solving ideas.

In VSM, the key people, resources, activities, and information flows involved in delivering a product or service, are drawn on a graph. VSM provides a deep understanding of how work is currently being performed and where work is unreliable or inconsistent. VSM is a key tool for identifying opportunities to reduce waste and integrate process steps more tightly, in order to improve process efficiency [8].

VSM does not just focus on single processes; but instead, creates a holistic schema of all processes and workflows, to understand the interdependency of functions and departments, and the effect of the unit as a whole [7]. Similarly, the A3 problem-solving method was originally borrowed from the Toyota Motor Company, and adapted to manufacturing companies in the United States and elsewhere. By reworking and removing workarounds, the A3 problem solving method improves the value stream.

Simply designing the map is insufficient for identifying waste. Once a VSM has been designed as a starting point, problems, such as long waiting times between steps or high amounts of reworking, can be identified and solved more easily. Tasks that do not add any value to the process (especially to the customers) should be removed before implementing any HIS solution. Therefore, identifying all of the value-added and non-value-added activities in the VSM, and assessing their impact on the overall process, is essential. Once the improvements in VSM have been identified, a future version of the VSM should be created, to show how the process will work if it is redesigned. This could include, for example, a smaller number of process steps and/or a shorter waiting time between steps [21].

In Lean application design, cultural changes need to be considered to achieve the desired result [10]. The importance of cultural changes has been reported by many researchers [10,22,23]. It is difficult for employees, particularly those who have been in their positions for a long time, to accept standardized work guidelines because they think that they already know how to perform the work correctly. However, employees will follow standardized work guidelines, when they understand the reasons behind them [21]. The Lean method is suitable for solving chronic problems, because it uses simple problem-solving tools, such as VSM and A3. A key advantage of the Lean transformation is in establishing a culture of continuous improvement and organizational learning. After the Lean method has been implemented, continuous improvement is important as a practice, because it allows for the monitoring and measuring of changes.

However, the Lean method has a number of limitations that need to be considered in its application. A fundamental limitation of one of its tools, VSM, is that it requires manual process mapping. Even with the aid of eVSM software, in designing these maps electronically, the basic map needs to be designed on paper during data collection. As described earlier, VSM needs to be complemented with other tools, such as A3 [9]. While VSM is used as a method for visualizing and understanding work from a high level view, A3 problem-solving uses the same logic, but focuses on specific problems with microscopic detail [24]. However, A3 is not applicable for very complex situations, because it focuses on finding the root cause of the problem. In a complex situation, the link between cause and effect is often unclear; therefore, A3 is not a suitable option for chaotic situations. In addition, there is no theoretical basis for generating an A3 report, which results in a variety of reports constructed by various analysts, who have differing subjective views on the same issues, thus leading to ambiguity and multiple interpretations [9].

There are a limited number of comprehensive studies on, and weak evidence for, Lean health care quality improvement; particularly those related to HIS [10,25]. Applying the Lean method in the clinical process, as related to HIS, helps us to obtain a more accurate view of the process and find errors and waste. We applied the Lean method and its related tools in an actual clinical setting, to reduce cycle time and remove waste.

## Methods

### Research methodology

We conducted a case study in order to have a better understanding of the clinical process related to the clinical care information system.

### Data collection

Data was collected by one of the authors using interview, observation, and document analysis methods. The observations were performed over sixteen working days in June, 2011. Clinical processes were observed for around eight hours each day, totalling 128 hours of observation time. We recorded the time frame for each step involved in the entire process of a pre-anaesthesia check-up. Ten semi-structured, face-to-face, individual interviews were conducted during fifteen to forty-five minute sessions. The interview questions are attached in Appendix A.

Site observations of the process allowed us to validate the flow of data from the beginning of the process to the end. For example, the anaesthetist is required to verify that the patient is ready for an operation by performing a physical assessment. However, the anaesthetist cannot start their assessments until after 3 pm, when the operation list is ready for the following day.

### Study location

The case study was performed in the Anaesthesia Department at the National Heart Institute (“Institut Jantung Negara”) (IJN) of Malaysia. Prior approval was obtained to conduct the study.

### Study subject and respondents

We investigated the use of a Critical Care Information Systems (CCIS) known as the IntelliVue Clinical Information Portfolio (ICIP) during the pre-anaesthesia process; particularly during the pre-anaesthesia physical assessment. The ICIP helps to streamline the workflow and provides support for automated regulatory and protocol compliance for the anaesthetists. The ICIP was commercially purchased from the Philips Company, to automate anaesthesia documentation and optimize patient care, throughput, and reimbursement. The ICIP was deployed mid-2009, two years before our case study was conducted, and no similar system had been launched before it.

The aim of the ICIP is to provide a paperless environment, as well as to improve patient care and safety, in an intensive care provision. It uses a centralized patient repository to manage and perform a number of functions, namely automatic charting, which includes vital signs, bedside device data, laboratories, pathology reports, medication orders, planned interventions, and other related data for nursing care and monitoring, anaesthesia care, surgical care, and allied health. In order to ease the technology adoption process, improve patient safety, and increase efficiency, the system is customized according to the IJN organizational setting.

**Figure 1:**
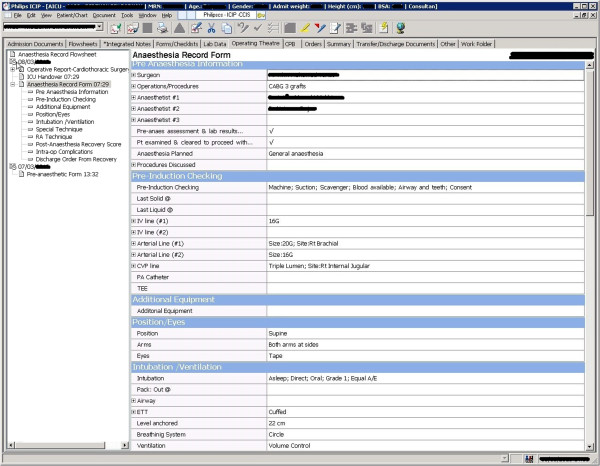
**CCIS’ ICU anaesthesia record form.**

All nurses are required to use the system, and all used it to chart and generate nursing progress notes and reports on a daily basis. Several doctors used the system for patient care planning and census generation from patients, while allied health professionals; particularly physiologists, used it to monitor patient progress. Figure 1 illustrates an example of the ICIP interface for the anaesthesia record form in the Intensive Control Unit (ICU).

We collected feedback from anaesthetists on their use of the ICIP and observed their interaction with the system. Ten participants were selected from those who were involved in the selected workflow. They consisted of four consultant anaesthetists, two anaesthetists, three clinical specialists, and one anaesthesia fellow, who was selected based on a purposive sampling method. An anaesthesia fellow is a trainee from another organization who spends one to two years at the IJN, while a clinical specialist is a qualified anaesthesiologist who is undergoing cardiac subspecialty training.

### Data analysis and analysis framework

The collected data was used to design the VSM and investigate the application of the ICIP in the clinical process. We used eVSM to map the stated clinical process (eVSM is a software tool that is designed to support maps and other visuals commonly leveraged in Lean implementations, such as value stream maps). We adopted the A3 tool to eliminate waste from the automated clinical workflow to achieve the desired outcome. eVSM also supports the A3 tool, which is used to design and map the “to-be” process.

### Validity and reliability of the research

In order to ensure the validity and reliability of the research, we triangulated our collected data from multiple sources and respondents. The designed process and the A3 map were then reviewed with the process owner, to validate our data in relation to the study's purpose.

## Results

Each anaesthetist has either completed or passed a short-term training course to use the ICIP, or has learned how to use the system, by working with another anaesthetist. All anaesthetists mentioned that the level of training was sufficient to work with the system. The system is sufficiently user-friendly and does not have any unidentified errors. All anaesthetists have access to the ICIP, and system usage can be tracked according to its user log history.

Prior to conducting this case study, the anaesthesia department performed an assessment on the usage rate of the ICIP, by checking the degree of completion of documents; particularly the pre-anaesthesia forms after operations. The assessment shows that in 2011, the percentages of filled in ICIP pre-anaesthesia forms were 44% in January, 45% in February, 34.6% in March, and 69.4% in April.

In terms of data completeness, approximately 40% of the patient demographic data was not entered into the system; therefore, the doctors had to complete the information themselves. All of the anaesthetists questioned the need for entering data from the lab and echocardiograph reports into the system, because this process is time-consuming and takes anaesthetists away from their other responsibilities. They argued that someone else should enter this information. This is a common reason why anaesthetists refuse to use ICIP during the pre-anaesthesia process. It is generally the responsibility of the anaesthetist to verify the accuracy of entered information pertaining to the anaesthesia process. However, they prefer to see verified data when they log in to the system.

Information that should be entered into the form includes the lab report, echocardiograph report, preoperative history, physical and airway examination, anaesthetic plan and risk, preoperative medication, and orders. This information must be made available during the patient’s visit. Most of the time, the information is ready - but not always.

The pre-anaesthesia process focuses on the activities related to anaesthesia that should be performed a day before each patient's operation. The anaesthetist records all necessary information during the patient’s physical assessment. This process includes the following steps: operating theatre list preparation, premedication process, patient education, anaesthesia consent, and anaesthesia planning and documentation. During this process, the anaesthetist sees all patients who are on the operation list, one day before surgery. During the patient’s visit, an anaesthetist performs a physical assessment of the patient and reviews the lab and echocardiograph reports to provide the patient with accurate medication. The pre-anaesthesia process starts when the operating theatre list is prepared. An anaesthetist collects the operation list for the following day from a nurse, before visiting the patients. The process can be delayed if there is an issue with the patient, lab reports, or finding a patient meeting room or Personal Computer (PC).

A PC, installed with the ICIP, is located in the patient’s meeting room for the anaesthetists to log onto and enter their comments and information on medication into the system. These PCs are occasionally used by nurses, because the number of PCs at the nurse’s stations is insufficient. Therefore, these situations can cause delays for the anaesthetists, in starting the physical assessment of the patients. Sometimes, the anaesthetists are unable to use the ICIP due to technical problems with the PC or the system. When problems occur, technical staff are usually unavailable to solve them. Anaesthetists are often too tired to wait for the system to resume, because most physical assessments are performed after 3 pm. In this case, they prefer to use paper instead of the ICIP.

In terms of patient availability, 10% of the patients were not ready when an anaesthetist went to the ward to visit them; thus requiring the anaesthetist to revisit them. During the patient’s revisits, the anaesthetists sometimes found that another anaesthetist had already completed the physical assessment without prior notification.

When the anaesthetist completes the pre-anaesthesia assessment form, the preoperative medication and orders should be adhered to by the nurse. The medication and its directions for use are written by the anaesthetist on paper for the nurse to follow. When the anaesthetist uses the ICIP instead of paper, all directions and medications are given to the nurse verbally, because the nurse does not have access to the ICIP. The outcome of this process is to ensure that the patient is physically ready for the operation, and that the anaesthetist’s instructions are followed one day before the operation.

In practice, anaesthetists do not follow a standard flow to complete their work. During our observations, only one out of ten anaesthetists used the ICIP when doing their job, while the others preferred to use a paper-based system.

In order to better understand the problems and propose a solution, we organized this paper as follows: a value stream map of the pre-anaesthesia process and A3 reports to find the root cause of the problem and our proposed future state map.

### VSM of the pre-anaesthesia process

**Figure 2:**
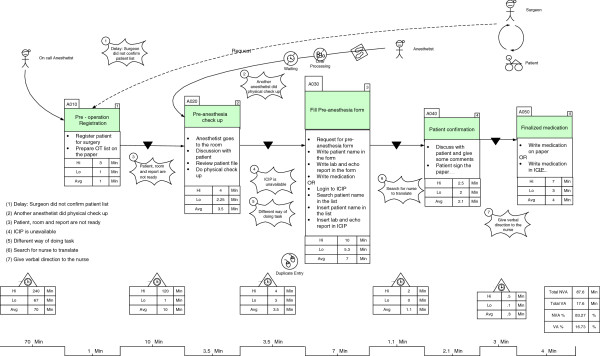
**Value Stream Map of pre**-**anaesthesia.**

The as-is VSM of the pre-anaesthesia process is depicted in Figure 2. When we consider the time taken to complete each step, as well as the delay between steps, we can identify the waste and defects of the as-is process. The non-value-added activities are represented in Figure 2 by deltas. A value stream map illustrates the way the actual work was performed and clearly highlights any delays that may occur. This delay enables us to determine how much of the total time spent in the process is value added to the patient, as opposed to being non-value-added. The lowest number in each box reflects the shortest amount of time required to complete the step being measured. Conversely, the highest number in each box shows the longest time required to complete the step being measured; thus highlighting an unusually complex set of activities, or one in which many interruptions or workarounds may have occurred. Again, the lowest number in the delta demonstrates the shortest delay between essential steps, and the highest number indicates the longest delay. Once we had identified the problems in the VSM, we proceeded to further analyse the problems. To find the root cause of each problem and remove it, we adopted a root cause analysis to determine the reasons for the problems using the A3 problem-solving method.

The table in Figure 2 shows the percentage of both value-added and non-value-added time for each step, as well as the time required to complete the entire process. The comparison of value-added time (the process boxes) and non-value-added time (the deltas) reveals a shocking discovery. We recorded the shortest and longest amounts of time required to complete the process for each step. Furthermore, we calculated the average time spent for each step, as well as the deltas based on the ten participants’ time spent on each step. The total non-value-added time in the pre-anaesthesia process, calculated based on the average time for each step, is 83.27% of the total time; which is very high. If we look at the VSM in detail, we can see the wasteful areas that caused the process delays.

The highest delay occurred at the start of the “pre-operation registration” step, with an average delay time of 70 minutes. The operation list should be ready by 2 pm on the day before the operation. However, surgeons are usually too busy performing in the OR to confirm the operation list for the following day; and therefore, the operation list is usually only available after 3 or 4 pm. If the list is not ready before 5 pm, the on-call anaesthetist will visit the patient to do a physical assessment and prescribe medication. Furthermore, the average delay between the “pre-operation registration” and the “pre-anaesthesia check-up” was 10 minutes, which caused the total non-value-added time to increase. Next, the average delay between the “pre-anaesthesia check-up” and the “fill in the pre-anaesthesia form” step was 3.5 minutes, which was due to duplicate data entries or the unavailability of the ICIP system because of technical problems. The total percentage of all value-added steps is 16.73% of the total time, which is clearly too low for this process. The waste that increased the total non-value-added time in the process is discussed in detail in the following subsection.

### Preanaesthesia current state map

In the as-is process of preanaesthesia, various types of waste and defects were identified. One of them was waiting waste (labelled as ‘number 1’ in Figure 2). The root cause of this waste was the waiting time for surgeon confirmations. Other identified types of waste were motion and unnecessary activities (labelled as ‘number 2’ in Figure 2). When anaesthetists go to the wards to visit patients, they sometimes find that another anaesthetist has already performed the physical assessment. This is due to a lack of task coordination, which leads to over-processing. We also identified a third type of waste i.e., the waiting time for resources, such as information, equipment, and systems (labelled as ‘number 3’ and ‘number 4’ in Figure 2). When an anaesthetist starts the physical assessment process, it is essential that the lab, echocardiograph, and lung function reports are ready and that the room and the patient are available. However, most of the time, at least one (if not all) of the above items are unavailable to implement the process. The unavailability of the system, due to a lack of computers in the paediatric ward, and technical problems occurring in PCs and the system is highlighted as ‘number 4’ in Figure 2.

During the patient visit, an anaesthetist can use two different ways to complete the pre-anaesthesia form (paper-based and ICIP), indicating a non-standardization of task implementation (labelled as ‘number 5’ in Figure 2). Next, we identified a communication problem, related to searching for a nurse to translate labelled as ‘number 6’ in Figure 2). Some foreign anaesthetists are unable to speak Malay (or English), but most of the patients are Malaysian.

We also identified confusion (with verbal medication directions to the nurse) as being another type of waste (labelled as ‘number 7’ in Figure 2). When the anaesthetists wrote directions and medication instructions on the pre-anaesthesia form in the ICIP, the completed form was not printed for inclusion in the patient’s history file. Because nurses do not have access to the ICIP, this situation resulted in verbal instructions being given; thus increasing the risk of medication errors. Task duplication was identified as originating from the data entry process. Multiple reports, such as lab, echocardiographs, and lung functions, were entered into different software systems, and the relevant information from those reports were entered again into the ICIP, resulting in data redundancy due to the lack of an integrated information system.

### Problem identification using A3

An A3 report is a storyboard that visualizes how a current process happens, what is wrong with it, and why it happens that way. These factors are illustrated on the left side of an A3 report. The right side of the report illustrates a better work process, the issues that need changing, and a plan for changing them.

**Figure 3:**
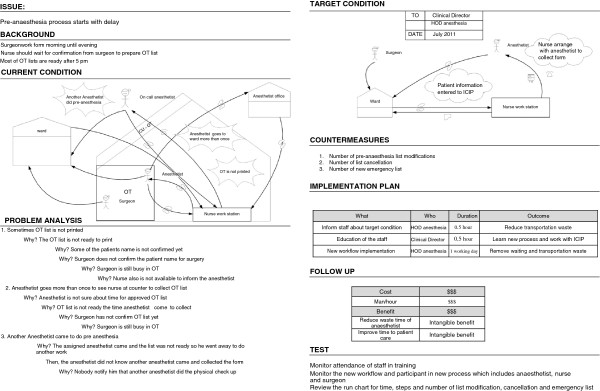
A3 Preanaesthesia (delay to start).

**Figure 4:**
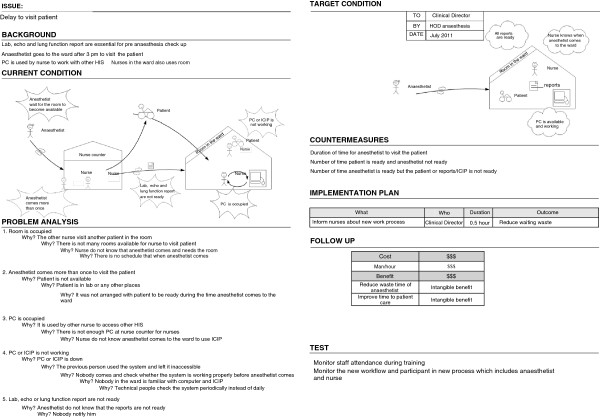
A3 Preanaesthesia (delay to visit patient).

**Figure 5:**
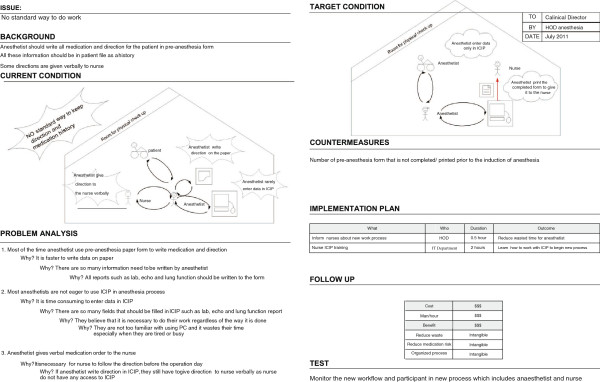
A3 Preanaesthesia (no standardized way).

**Figure 6:**
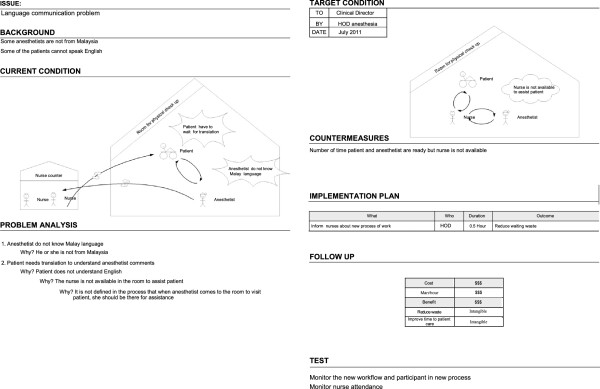
A3 Preanaesthesia (interaction problem).

We identified seven problems in the pre-anaesthesia process. When we analysed the root cause of these problems, we found that some of them had similarities. We therefore developed four A3 reports that cover the aforementioned problems, namely the delay in the start of the pre-anaesthesia process, the delay in patient visits, the non-standardized working process, and the interaction problem. For each problem, we developed an A3 report to perform a root cause analysis, in order to design an improved process (see Figures 3, 4, 5, and 6).

## Discussion

The anaesthesia department performed an assessment of the anaesthetist’s use of ICIP by checking the completion of documents; particularly the pre-anaesthesia form, before this case study was performed. The assessment shows that in 2011, the percentages of filled in ICIP pre-anaesthesia forms were 44% in January, 45% in February, 34.6% in March, and 69.4% in April. The completion of the pre-anaesthesia form is assessed after the operation, based on the availability of the printed form in the patient’s file, and not on the completion of the form. Many of the anaesthetists completed the form, but did not print it out to keep in the patient’s file. Furthermore, several anaesthetists completed the form after the operation; instead of one day before. Therefore, these assessment results were inaccurate, because the assessment was performed after operations, and there is no evidence to show whether anaesthetists completed the necessary forms in the ICIP before or after each operation. The inaccuracy of these assessment results is also affected by a lack of availability of the ICIP system in the paediatric department, meaning that anaesthetists are unable to complete the pre-anaesthesia form during patient visits.

Since the results of the previous assessment were inaccurate, and no performance measurements of the current system were performed, an evaluation of the clinical process was needed to determine the best possible performance, in terms of removing waste and increasing the process flow. Therefore, we investigated the pre-anaesthesia process related to the ICIP and focused more on the workflow to remove defects and waste.

In order to evaluate the existing process related to the ICIP, we identified the problems and classified them to find their root causes. We classified these problems from three intertwined factors: Human, Organizational, and Technology (HOT); and the fit between them, based on the HOT-fit evaluation framework [26]. Human, organization and technology factors are the essential components of any IS, and the impact of any HIS system is assessed by evaluating its net benefits. These three factors, and their impact on HIS, correspond to the nine interrelated dimensions of HIS success: technology (system quality, information quality, and service quality), human (system development, system use, and user satisfaction), organization (organizational structure and organizational environment), and net benefits. Each of these dimensions is associated with a number of evaluation measures.

In terms of the technology factor, we identified three problems from the System Quality dimension: system availability, flexibility, and reliability. The unavailability of the ICIP in the paediatrics’ department is one of the reasons why the anaesthetists are not eager to use the ICIP to complete the pre-anaesthesia form. In terms of flexibility, the information and the database between the different platforms are not integrated or linked. For example, the specified software for the lab report is not integrated in the ICIP. As a result, the printed lab reports need to be manually attached to the patient’s file. All critical information, relating to the patient, needs to be recorded in the ICIP; therefore, the anaesthetist must enter this data manually into the ICIP, so that it can be accessible in the operating room. Technical problems are either hardware or software related. We also identified a number of problems from the technology dimension of Information Quality, namely: fragmented information, late reporting, repetitive information, incomplete data entry, and delay of lab reports.

In terms of human factors, we identified user knowledge and skill problems from the System Use dimension. Users lacked a basic knowledge of the operating system, and their slow typing speed affected their task when entering data into the ICIP. In terms of organizational factors, problems were identified in the Organizational Structure (i.e., the internal parts of the organization): resources, clinical process, and a culture that caused process delays. A shortage of resources was identified for the pre-anaesthesia procedure, in terms of technical staff and room availability. Moreover, there was a lack of integration between the people involved in the clinical process. Every person works in a different way, which means there is no standardized work method in the process. It would be difficult to change the way clinicians perform their work using the new HIS, because anaesthetists prefer to use a paper-based system. Even in their best form, paper documents have legibility and accuracy issues [27]. Manual transcription is also time-consuming, and in complex cases, the number of parameters that need to be documented may increase greatly; and may create a possible time lag between observation and charting.

These problems cause the people who are involved in the process to ignore the role of the ICIP in their work. Furthermore, when the information is not integrated between various related departments, it increases the human error factor in data entry. When the activities that are included in one process are not standardized, the need for verbal directions for medication increases and the use of the ICIP decreases. The lack of standardization also caused more delays in the process and increased waste. As our focus in this research was to apply the Lean method, to clinical processes that are related to HIS, we focused more on the workflow to identify waste and remove defects from the process, that were directly related to ICIP.

### Study limitations

This study has three limitations. First, the study scope is small as it is limited to one specific clinical process only. Hence, the findings may not represent the overall clinical process, other users, and system use. However, our results can serve as an outlook that reports the feasibility of applying the Lean method in the HIS context. Second, the study is conducted in a specific tertiary healthcare setting. However, like Cima [13], our findings show that the Lean method can be applied to a dynamic, high-volume surgical practice, in order to identify wastes and their relevant improvements. Third, the study is conducted on a relatively short-term duration, capturing limited data types, without detailed statistical reports. Thus, this study was not able to demonstrate actual improvements to particular aspects of the exercise that reflected the multi-attribute nature of the Lean method [25].

The next part of this study discusses the existing clinical processes and related activities that could reduce waste and increase value-added activities.

### Pre-anaesthesia future state map (improved process model)

We attempted to simplify processes using as few steps as possible. When examining the steps, the number of connections between people provided a clear indication of the complexity of the process. Ideally, all connections should be as direct as possible, with the fewest steps and the fewest people being involved in relaying the process.

**Figure 7:**
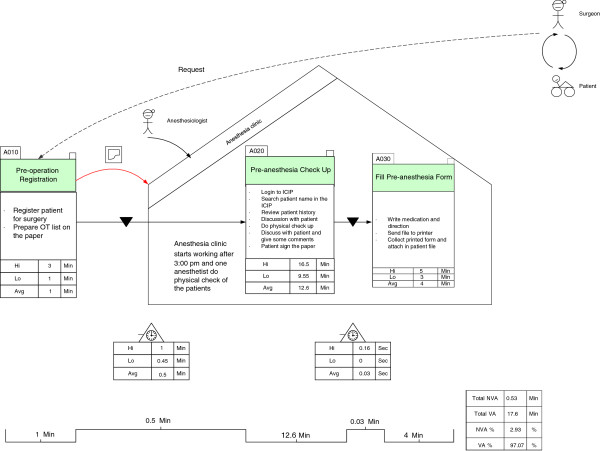
**To-be Preanaesthesia process.**

We removed waste and problems from the pre-anaesthesia process and subsequently developed a future state map using the A3 problem-solving method. In the future state map, we suggested that the hospital conduct a pre-anaesthesia clinic, which, depending on the workload, would assign one or more anaesthetists to visit patients and prepare the pre-anaesthesia reports, with the goal of removing over-processing waste. Given that anaesthetists work in the operating room from morning until late afternoon, it is vital for them to focus more on their core tasks. The quality and efficiency of preoperative care is likely to be affected when anaesthetists need to allocate time for accessing multiple data sources and Clinical Information Systems (CIS), and when they depend on manually documenting information in various systems [28]. In this proposed solution, the anaesthetists in charge of physical assessments, would be available at the pre-anaesthesia clinic and could concentrate on their tasks. The future state map is shown in Figure 7, along with the suggested changes for removing waste from the current state map.

The highest delay identified, in the pre-anaesthesia process, occurred at the start of the “pre-operation registration” step, with an average delay of 70 minutes. These delays would not appear in the future state map, if anaesthetists started their work with the first patient who was ready for physical assessment. The over-processing waste, identified in the current process, can also be removed if there is only one anaesthetist in charge of performing patient physical assessments.

In order to resolve duplicate data entries, we proposed that the ICIP be integrated with the Electronic Medical Record (EMR) and other relevant HIS and devices. The actual advantages of an Anaesthesia Information Management System (AIMS) (which in our case is part of the ICIP) can only be realized if it is fully integrated with other health IT systems or if it is able to communicate automatically and bidirectionally with them [28,29]. There are a number of options that can be chosen in integrating AIMS with EMR [29,30]. An enterprise EMR enables the merging of anaesthesia documentation with other clinician documentation, as well as the ability to interface with related medical devices [30]. With this proposal, the current process that requires anaesthetists to enter lab, echocardiograph, and lung function reports, will be eliminated. Under the proposal, when anaesthetists go to the anaesthesia clinic, they can simply find this patient information in the ICIP without having to enter it, and thereby reduce the patient’s waiting time. As a result, the average delay between the “pre-operation registration” and the “pre-anaesthesia check-up” (of 10 minutes) can be removed from the current state map. This small change removes waiting waste from the process and reduces the process’s complexity. Furthermore, the integrated information may encourage anaesthetists to use the ICIP when performing their tasks, even if they were initially reluctant to use the ICIP before. As mentioned previously, most anaesthetists complained about having to perform data entry in the ICIP. They stated that it is time-consuming for them, and that their responsibility is to perform physical assessments, and not to work as a secretary.

Furthermore, with an integrated HIS, the ward nurse can check the availability of all reports that are required for the anaesthetist. When the anaesthetist goes to the clinic, they can login to the ICIP and read the relevant reports. This process can therefore reduce patient waiting time, as opposed to the as-is process, where anaesthetists must complete the form in the ICIP themselves, which keeps the patient waiting. Ideally, most medical history and physical assessment information should be collected from other sources and given to the anaesthetist for review [29]. The anaesthetist should spend more time analysing the information and developing an anaesthetic plan and less time collecting information.

Another suggested change in the future state map is process standardization. In the as-is process, anaesthetists can use either paper or the ICIP to write medication instructions and directions. In the to-be process, all anaesthetists can conveniently use the ICIP to complete the form, because all necessary data entered into the ICIP is linked to other systems through an integrated HIS. Reports can be printed, appended to a patient’s file, and referred to by the nurses, and thus confusion waste can be removed from the future state map. The standardization of preoperative evaluations, amongst other factors, has yielded a significant improvement in starting surgeries on time [13].

In the to-be process, the proposed solution is to conduct a daily anaesthesia clinic at 3 pm. The patient, the examination room, and all necessary equipment needed by the anaesthetists, can be arranged by nurses in advance, and the stated waiting waste can be removed from the future state map. Standardized room allocation guidelines were one of the factors that resulted in improved efficiency of the overall specialties operation of an academic medical centre [13]. As for the communication problems, we suggest that a nurse should be available in the examination room to assist foreign anaesthetists during patient visits, in order to eliminate the wasted miscommunication waiting time.

In addition to the above suggestions, a balance of quick fixes and more fundamental approaches, such as a “well-coordinated and planned multidisciplinary approach,” could reduce waste and subsequently improve the quality of care; as demonstrated by Yousri et al., [11]. Mazzocato et al., [25] showed improvements in emergency care by adopting this approach, based on four Lean rules [31]: standardized work and reduced ambiguity, linked interdependent people, enhanced processes that are seamless and uninterrupted, and empowered staff to identify problems and make improvements using a scientific method.

Based on our suggestions, we calculated the total value-added and non-value-added time of the process in the designed future state map. As shown in Figure 7, the value-added time increased to 97.07% of the total time, and the non-value-added time decreased to 2.93%. In order to achieve the future state map, the implementation plan provides a structure to improve work and its required cost, time, and effort. Calculating the cost benefit provides a fair justification for the cost of the plan. However, monetary measures may be reflected later in patient safety factors, quality of care, patient satisfaction, and workplace appreciation.

**Figure 8:**
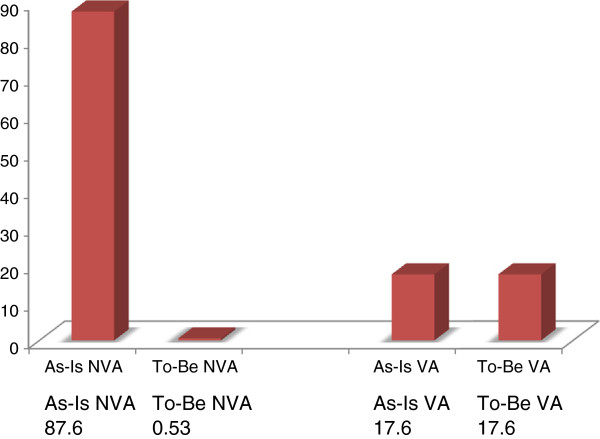
Comparison of total VA and NVA of Preanaesthesia process.

In summary, we designed a chart based on a timeframe analysis of the as-is and to-be processes of value-added and non-value-added activities, in order to identify improvements for each studied process (see Figure 8). When we compared the results based on the timeframe, it is obvious that the total non-value-added time decreased in all processes. Figure 8 shows that the total non-value-added time in the to-be process decreased, because in the current process, there were activities that were considered as waste. Therefore, by removing those activities, we simplified the overall process flow.

## Conclusions

We used the Lean method to increase collaboration and teamwork across departments to minimize the risk of sub-optimization, which we demonstrated in the future state map. We also assessed and redesigned the automated workflow and recommended an integrated clinical information system to increase database consistency and data completeness. We applied the Lean method to analyse the problem by exploring a root cause analysis and identifying both value-added and non-value-added time. Based on the analysis, we recommended improvements, in terms of increased collaboration and teamwork. Furthermore, we proposed a standardized way of performing tasks by encouraging anaesthetists to use ICIP, based on its integration with HIS. We applied the Lean method as a tool to help us improve effectiveness and increase the quality of healthcare delivery by aligning the process, the people, and the technology, with each other.

This study was performed to assess the feasibility of implementing the Lean method using VSM and A3 tools in evaluating the clinical process. We believe that there is still more work that needs to be performed and tangible measures that need to be instituted into the initiative, before any clear demonstrable benefit can be shown. However, the study has achieved its purpose in demonstrating that the application of the Lean method; particularly the feasibility of using both the VSM and A3 tools, is an effective mechanism for identifying opportunities for reducing waste, for integrating process steps more tightly, and for standardizing work in the clinical process. Nevertheless, for this proposed solution to succeed, it needs to be implemented enterprise-wide and with extensive training and knowledge acquisition.

## Appendix A: Interview Questions

1. How does a new anaesthetist learn this process to work with the ICIP?2. Were the anaesthetists trained properly in the use of the software?3. Do they have access to easy-to-use documentation that is written in a very simple language?4. Is there any limitation in using the software to enter the patient information?5. Does the software display any errors or unknown messages during data entry?6. When is the operation list usually ready?7. Is the PC used for other purposes?8. Is the ICIP software available any time they want to use it in the ward?9. Who is responsible for the information that is entered in the pre-anaesthesia form?10. What information is required to fill in the form?11. Is all of the information available when the anaesthetist visits the patient to fill in the form?12. How many fields are mandatory?13. Is the current condition ideal?14. Does the work happen in a straight line with a continuous flow?15. What roadblocks exist that must be worked around?16. Are all of the steps in the process necessary?17. Is the information flow direct and simple?18. Is the participation of every person who touches the process necessary?19. Does everyone who is involved in the process work the same way?

## Competing interests

The authors declare that they have no competing interests.

## Authors’ contributions

MMY suggested the subject. AMBM suggested the area of the case study at the National Heart Institute. SKH conducted the case study and reported the findings under the close supervision of MMY and AMBM. AMBM was the clinical supervisor. MMY was the research supervisor. SKH drafted the paper. MMY approved and finalized it. All authors read and approved the final manuscript.

## Pre-publication history

The pre-publication history for this paper can be accessed here:

http://www.biomedcentral.com/1472-6947/12/150/prepub
